# Supplementary material for: The Inhibition of Complement System in Formal and Emerging Indications: Results from Parallel One-Stage Pairwise and Network Meta-Analyses of Clinical Trials and Real-Life Data Studies
Source: Biomedicines. 2020 Sep 16;8(9):355. doi: 10.3390/biomedicines8090355 (PMC7554929; doi:10.3390/biomedicines8090355)
Supplement: Supplementary file 1 [file biomedicines-08-00355-s001.pdf]

Supplementary material of the article:

The inhibition of complement system in formal and emerging indications: Results from parallel one-stage pairwise and network meta-analyses of clinical trials and real-life data studies

Coralina Bernuy-Guevara , Hassib Chehade , Yannick D. Muller , Julien Vionnet , François Cachat , Gabriella Guzzo , Carlos Ochoa-Sangrador , F. Javier Álvarez , Daniel Teta , Débora Martín-García , Marcel Adler , Félix J. de Paz , Frank Lizaraso-Soto , Manuel Pascual , Francisco Herrera-Gómez

Table S1: The included studies after searches with respect to (a) their relevant information from study participants grouped by diseases, (b) the risk of bias in clinical trials, and (c) the risk of bias in real-life NRSI.

Figure S1: Forest and inconsistency plot for (a) hemolysis in PNH, (b) TMA in aHUS, and (c) AKI in aHUS.

Panel S1. Full search strategy and search results.

Table S1. The included studies after searches with respect to:
(a) Their relevant information from study participants grouped by diseases,

Information from clinical trial (R/NR)[€] / NRSI study in:	Study participant characteristics:
**PNH**	Age (range in yr), AA/MDS (%), disease duration (range in yr), previous/concomitant treatments (%):
(a) Eculizumab parent studies (1/2) & (b) common extension study (0/1)	18–85, 30.3, 0–39, 36.0
(c) AEGIS (0/1) plus extension follow-up	26–70, 45.0, NA, 48.0
(d) 301 (1/0) plus extension follow-up	30–61, NA, 3–4, NA
(e) 302 (1/0) plus extension follow-up	34–62, 37.4, 3–21, NA
(f) International PNH registry & (g) country PNH registries[$]	3–99, 21.0, 1–47, 28.0
**aHUS**	Age (range in yr), disease duration (range in mo), previous/concomitant treatments (%), KRT need (%):
(h) C08-002 (0/1) plus extension follow-up	17–68, 0–236, 100.0, 35.0
(i) C08-003 (0/1) plus extension follow-up	13–63, 1–286, 100.0, 10.0
(j) C10-003 & (k) C10-004 (0/2)	0–80, 0–313, 100.0, 41.0
(l) C09-001r (0/1) & common extension study for eculizumab parent studies (0/1)	0–80, 0–313, 100.0, 39.0
(m) 311 (0/1)	18–79, NA, 100.0, 45.0
(n) Global aHUS registry & (o) country aHUS registries[$]	0–82, 0–612, 100.0, 47.0
**gMG**	Age (range in yr), disease duration (range in yr), previous/concomitant treatments (%):
(p) Eculizumab pilot study (1/0)	30–72, 1–30, 50.0
(q) REGAIN (1/0) & ECU-MG-302 (0/1)	20–57, 1–18, 96.0
**aAMR**	Age (range in yr), allo-reactivity findings (%), post-TX time (range in yr), previous/concomitant treatments (%):
(r) Berinert pilot study (0/1)[£]	44–58, 100.0, 0–16, 100.0
(s) Cinryze pilot study (1/0) & post-hoc analysis[£]	36–61, 44.0, 70–366, 78.0
(t) 07-007208 (0/1)[£]	37–61, 100.0, 0.0, 69.0
(u) Berinert exploratory study (1/0) & post-hoc analyses[£]	32–62, 40.0, 0.0, 100.0
(v) C10-001 (1/0)[£]	29–57, 100.0, 0.0, 100.0
(w) C10-002 (0/1) plus extension follow-up[£]	24–70, 100.0, 0.0, 0.0
**DGF**	Age (range in yr), risk for DGF (%), previous/concomitant treatments (%):
(x) Eculizumab for reperfusion (1/0) plus extension follow-up	31–51, 30.0, 100.0
(y) C1INHDGF (1/0) plus extension follow-up	48–67, 42.0, 100.0
(z) 10-1600 & 13-0920 (2/0)	51–68, 100.0, 100.0
(aa) PROTECT (1/0) plus extension follow-up	18–55, 100.0, 100.0

(b) the risk of bias in clinical trials,

**Trials**	**Random sequence generation**	**Allocation concealment**	**Blinding of participants and personnel**	**Blinding of outcome assessment**	**Incomplete outcome data**	**Selective reporting**	**Other bias**
Pilot PNH (a)	H	H	H	H	H	H	U
TRIUMPH (a)	L	L	L	U	U	U	U
SHEPHERD (a)	H	H	H	H	U	U	U
PNH eculizumab parent studies common extension (b)	H	H	H	H	L	L	U
AEGIS (c)	H	H	H	H	H	H	U
301 (d)	L	L	U	U	U	U	U
302 (e)	L	L	U	U	U	U	U
C08-002 (h)	H	H	H	H	U	U	U
C08-003 (i)	H	H	H	H	U	U	U
C10-003 (j)	H	H	H	H	H	H	U
C10-004 (k)	H	H	H	H	H	H	U
C09-001r & C11-003 (l)	H	H	H	H	H	H	H
311 (m)	H	H	H	H	U	U	U
pilot rgMG (p)	L	L	L	U	U	U	H
REGAIN & extension (q)	L	L	L	U	H	H	U
Berinert pilot (r)	H	H	H	H	H	H	U
Cinryze pilot (s)	L	L	L	U	U	U	U
07-007208 (t)	H	H	H	H	H	H	U
Berinert exploratory study (u)	L	L	L	U	U	U	U
C10-001 (v)	L	L	L	U	U	U	U
C10-002 (w)	H	H	H	H	H	H	U
Eculizumab for reperfusion (x)	L	L	U	U	H	U	U
C1INHDGF (y)	L	L	L	U	U	U	U
10-1600 (z)	L	L	L	U	U	L	U
13-0920 (z)	L	L	L	U	U	L	U
PROTECT (aa)	L	L	L	L	U	U	U

(c) The risk of bias in real-life NRSI.

Real-life NRSI	Confounding	Participants selection	Interventions	Deviations	Missing data	Outcomes measurement	Selection of the reported result
International PNH registry (a)	S	M	S	S	C	M	S
Country PNH registries (a)	S	C	S	C	C	S	S
Global aHUS registry (a)	S	M	S	S	C	M	S
Country aHUS registries (b)	S	C	S	C	C	S	S

(a) The PNH eculizumab parent studies were the following: 12-week, single arm, open-label, phase 2 pilot study and its 1-year extension follow-up study (1, 2), 26-week, randomized, double-blind, placebo-controlled, phase 3 TRIUMPH study (NCT00122330) (3), and 52-week, single arm, open-label, phase 3 SHEPHERD study (NCT00130000) (4).
(b) 102-week, single arm, open-label, phase 3 common pilot-TRIUMPH-SHEPHERD extension study (5–7).
(c) 12-week, single arm, open-label, phase 2 pivotal AEGIS study (8) and its 2-year extension follow-up study (9).
(d) 26-week, randomized, open-label, active-controlled, phase 3 ALXN 1210-PNH-301 study (NCT02946463) and its 2-year extension follow-up study (10).
(e) 26-week, randomized, open-label, active-controlled, phase 3 ALXN 1210-PNH-302 study (NCT03056040) and its 2-year extension follow-up study (11).
(f) The International PNH registry (NCT01374360) was a global, prospective, non-interventional, observational study (12–16).
(g) The following country PNH registries were non-interventional, observational studies involving prospective (Japan (17) and Korea (18–21)) and retrospective (France (22, 23), Spain (24–26), Taiwan (27), and UK (28)) data collection.
(h) 26-week, single arm, open-label, phase 2 C08-002 study of Eculizumab (NCT00844545, NCT00844844) and its 2-year extension follow-up study (29–31).
(i) 26-week, single arm, open-label, phase 2 C08-003 study of Eculizumab (NCT00838513, NCT00844428) and its 2-year extension follow-up study (29–31).
(j) 26-week, single arm, open-label, phase 2 C10-003 study of Eculizumab (NCT01193348) (32).
(k) 26-week, single arm, open-label, phase 2 C10-004 study of Eculizumab (NCT01194973) (33).
(l) Retrospective, observational, C09-001r study of Eculizumab (NCT01770951) and the long-term prospective, observational, C11-003 follow-up study for all participants in Eculizumab parent studies C08-002, C08-003, C10-003, C10-004, and C09-001r (NCT01522170) (34, 35).
(m) 26-week, single arm, open-label, phase 3 ALXN 1210-aHUS-311 study (NCT02949128) (36).
(n) The Global aHUS registry (NCT01522183) was a global, prospective, non-interventional, observational study (37–40).
(o) The following country aHUS registries were non-interventional, observational studies involving prospective (Japan (41–43), Australia (44), and Brazil (45)), prospective/retrospective (Turkey (46–48)) and retrospective (France (49–55), Spain (56–58), and UK and Ireland (59, 60)) data collection.
(p) 37-week, crossover, randomized, double-blind, placebo-controlled, phase 2 pilot study of Eculizumab in rgMG (61).
(q) 24-week, randomized, double-blind, placebo-controlled, phase 3 REGAIN study (NCT01997229) (62) and its 2-year extension ECU-MG-302 study (NCT02301624) (63).
(r) 24-week, single arm, open-label, phase 2 pilot study of Berinert for treatment of acute AMR (64).
(s) 2-week, randomized, double-blind, placebo-controlled, phase 2 pilot study of Cinryze for treatment of acute AMR (NCT01147302) and its 6-month post-hoc analysis (65).
(t) 12-week, single arm, open-label, phase 2 07-007208 study of Eculizumab for prevention of AMR (NCT00670774) (66).
(u) 24-week, randomized, double-blind, placebo-controlled, phase 1/2 exploratory study of Berinert for prevention of AMR (NCT01134510) (67) and its 2-year and 3-year post hoc analyses (68, 69).
(v) 9-week, randomized, open-label, standard of care-controlled, phase 2 C10-001 study of Eculizumab for prevention of AMR (NCT01399593) (70).
(w) 9-week, single arm, open-label, phase 2 C10-002 study of Eculizumab for prevention of AMR (NCT01567085) and its 1-year and 3-year extension follow-up studies (71).

(x) 4-week, randomized, open-label, placebo-controlled, phase 2 study of Eculizumab for prevention of reperfusion injury (NCT01756508) and its 1-year and 3-year extension follow-up studies (72).
(y) 4-week, randomized, double-blind, placebo-controlled, phase 1/2 C1INHDGF study of Berinert for prevention of DGF (NCT02134314) (73) and its 1-year extension follow-up study.
(z) 24-week, randomized, double-blind, placebo-controlled phase 2 10-1600 pilot study of Eculizumab for prevention of DGF (NCT01403389), and 24-week, randomized, double-blind, placebo-controlled phase 2 13-0920 study of Eculizumab for prevention of DGF (NCT01919346) (74).
(aa) 26-week, randomized, double-blind, placebo-controlled, phase 2/3 PROTECT study (NCT02145182) and its 1-year extension follow-up study (75).

[€]For each study and in the case of more than one study, random allocation in a given study and the number of randomized and non-randomized trials in each group of studies are specified. [$]Until 83% and 75% out of data in country PNH registries (29–41) and country aHUS registries (54–73) were already considered, respectively, into the data of the International PNH registry (24–28) and the Global aHUS registry (50–53). [£]Two studies assessed complement inhibitors for uses as treatment (76, 77), and not for prevention of aAMR (78–83). [¥]Number of individuals undergoing the interventions or exposed to comparisons. [@]The judgement for each of risk of bias domain in the Cochrane Collaboration tool is presented as (L), (U) or (H) to indicate low, unclear, or high risk of bias, respectively. [@]The judgement for each of risk of bias domain in the ROBINS-I tool is presented as (L), (M), (S), (C), or (N) to indicate low, moderate, serious, critical, or no information on risk of bias, respectively.

Abbreviations: AA, aplastic anemia; AEGIS, AEGIS registration study of Eculizumab in Japanese patients with paroxysmal nocturnal hemoglobinuria; aHUS, atypical hemolytic uremic syndrome; AMR, antibody-mediated rejection; C1INHDGF, C1INH (Berinert) for DGF study; DGF, delayed graft function; ECU-MG-302, extension study of ECU-MG-301 to evaluate safety and efficacy of Eculizumab in refractory generalized myasthenia gravis; ICA-GBS, Inhibition of Complement Activation in Guillain-Barré syndrome; JET-GBS, Japanese Eculizumab Trial for Guillain-Barré syndrome; MDS, myelodysplastic syndrome; PNH, paroxysmal nocturnal hemoglobinuria; PROTECT, Study of Eculizumab for the prevention of delayed graft function after kidney transplantation; REGAIN, safety and efficacy of eculizumab in REfractory GenerAlIzed MyastheNia Gravis; rgMG, refractory generalized myasthenia gravis; ROBINS-I, Risk Of Bias In Non-randomized Studies - of Interventions; SHEPHERD, Safety in HEmolytic PnH patients treated with Eculizumab: a multi-center open-label Research Design; TRIUMPH, Transfusion Reduction efficacy and safety clinical Investigation, a randomized, multicenter, double-blind, placebo-controlled, Using eculizuMab in Paroxysmal nocturnal Hemoglobinuria.

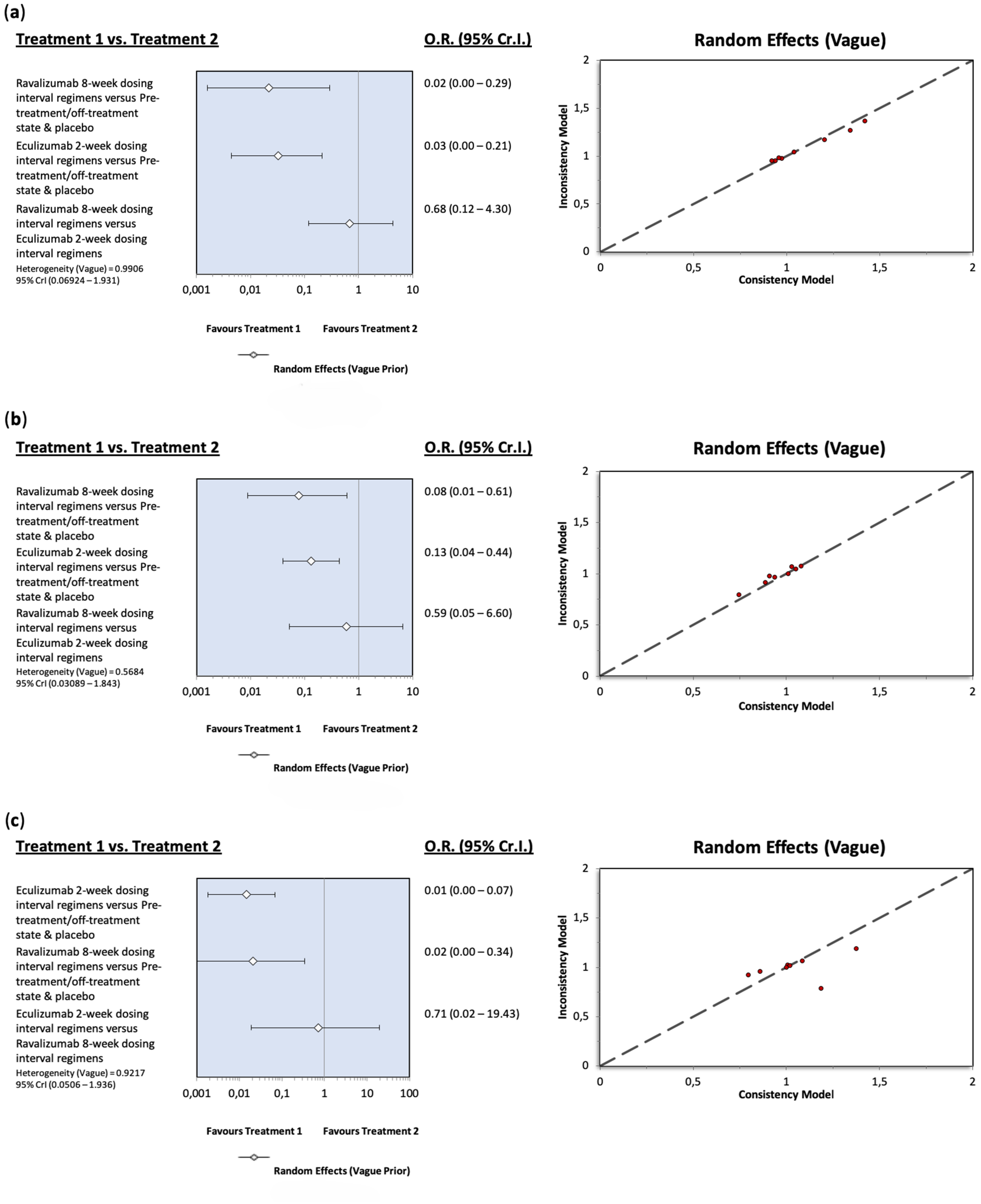

Figure S1. Forest and inconsistency plot for (a) hemolysis in PNH and (b) TMA and (c) AKI in aHUS. aHUS; atypical hemolytic uremic syndrome; AKI; acute kidney injury; PNH, paroxysmal nocturnal hemoglobinuria; TMA, thrombotic microangiopathy

Panel S1. Full search strategy and search results.

Formulae	Citations
**PubMed**	
(((("hemoglobinuria, paroxysmal"[MeSH Terms]) OR "atypical hemolytic uremic syndrome"[MeSH Terms]) OR "myasthenia gravis"[MeSH Terms]) OR "graft rejection"[MeSH Terms]) OR "delayed graft function"[MeSH Terms]	79553
((((("paroxysmal nocturnal hemoglobinuria"[Text Word]) OR "atypical hemolytic uremic syndrome"[Text Word]) OR "myasthenia gravis"[Text Word]) OR "antibody mediated rejection"[Text Word]) OR "ischemia reperfusion injury"[Text Word]) OR "delayed graft function"[Text Word]	48752
("eculizumab"[Supplementary Concept]) OR "ravulizumab"[Supplementary Concept]	930
("eculizumab"[Text Word]) OR "soliris"[Text Word]	1663
(("ravulizumab"[Text Word]) OR "ultomiris"[Text Word]) OR "alxn1210"[Text Word]	19
"complement c1 inhibitor protein"[MeSH Terms]	1091
("serping1 protein, human"[Supplementary Concept]) OR "conestat alpha"[Supplementary Concept]	365
(((("c1 esterase inhibitor"[Text Word]) OR "berinert"[Text Word]) OR "cinryze"[Text Word]) OR "haegarda"[Text Word]) OR "ruconest"[Text Word]	1041
**Ovid Medline(R)**	
exp hemoglobinuria, paroxysmal/ or exp atypical hemolytic uremic syndrome/ or exp myasthenia gravis/ or exp graft rejection/ or exp delayed graft function/<br>OvidMEDLINE(R) ALL 1946 to September 30, 2019	79540
(paroxysmal adj nocturnal adj hemoglobinuria).tw. or (atypical adj hemolytic adj uremic adj syndrome).tw. or (myasthenia adj gravis).tw. or (antibody adj mediated adj rejection).tw. or (ischemia adj reperfusion adj injury).tw. or (delayed adj graft adj function).tw.<br>Journals@Ovid<br>Subscribed Ovid journals by Sacyl<br>Ovid MEDLINE(R) ALL 1946 to September 30, 2019	113695
(Eculizumab or soliris).tw.<br>Journals@Ovid<br>Subscribed Ovid journals by Sacyl<br>Ovid MEDLINE(R) ALL 1946 to September 30, 2019	4552
(ravulizumab or ultomiris or alxn1210).tw.<br>Journals@Ovid<br>Subscribed Ovid journals by Sacyl<br>Ovid MEDLINE(R) ALL 1946 to September 30, 2019	41
exp complement c1 inhibitor protein/<br>OvidMEDLINE(R) ALL 1946 to March 31, 2020	1089
(c1 adj esterase adj inhibitor).tw. or berinert.tw. or cinryze.tw. or haegarda.tw. or ruconest.tw.<br>Journals@Ovid<br>Subscribed Ovid journals by Sacyl<br>Ovid MEDLINE(R) ALL 1946 to September 30, 2019	3181
**Elsevier's Scopus**	
(KEY ("paroxysmal nocturnal hemoglobinuria") OR KEY ("hemolytic uremic syndrome") OR KEY ("myasthenia gravis") OR KEY ("acute graft rejection") OR KEY ("antibody mediated rejection") OR KEY ("delayed graft function")) AND NOT INDEX (medline)	8891
(TITLE-ABS-KEY ("paroxysmal nocturnal hemoglobinuria") OR TITLE-ABS-KEY ("atypical hemolytic uremic syndrome") OR TITLE-ABS-KEY ("myasthenia gravis")	13143

OR TITLE-ABS-KEY ("antibody mediated rejection") OR TITLE-ABS-KEY ("ischemia reperfusion injury") OR TITLE-ABS-KEY ("delayed graft function")) AND NOT INDEX (medline)	
(KEY (eculizumab) OR KEY (ravulizumab)) AND NOT INDEX (medline)	729
(TITLE-ABS-KEY (eculizumab) OR TITLE-ABS-KEY (soliris)) AND NOT INDEX (medline)	788
(TITLE-ABS-KEY (ravulizumab) OR TITLE-ABS-KEY (ultomiris) OR TITLE-ABS-KEY (alxn1210)) AND NOT INDEX (medline)	5
KEY ("complement component C1s inhibitor") AND NOT INDEX (medline)	672
(KEY ("SERPING1 protein,human") OR KEY (haegarda)) AND NOT INDEX (medline)	14
(TITLE-ABS-KEY ("c1 esterase inhibitor") OR TITLE-ABS-KEY (berinert) OR TITLE-ABS-KEY (cinryze) OR TITLE-ABS-KEY (haegarda) OR TITLE-ABS-KEY (ruconest)) AND NOT INDEX (medline)	337
**Web of Science**	
TOPIC: ("paroxysmal nocturnal hemoglobinuria") OR TOPIC: ("atypical hemolytic uremic syndrome") OR TOPIC: ("myasthenia gravis") OR TOPIC: ("antibody mediated rejection") OR TOPIC: ("ischemia reperfusion injury") OR TOPIC: ("delayed graft function")<br>Web of Science Core Collection<br>Current Contents Connect<br>Derwent Innovations Index<br>KCI-Korean Journal Database<br>Medline<br>Russian Science Citation Index<br>SciELO Citation Index	101709
TOPIC: (eculizumab) OR TOPIC: (soliris)<br>Web of Science Core Collection<br>Current Contents Connect<br>Derwent Innovations Index<br>KCI-Korean Journal Database<br>Medline<br>Russian Science Citation Index<br>SciELO Citation Index	3428
TOPIC: (ravulizumab) OR TOPIC: (ultomiris) OR TOPIC: (alxn1210)<br>Web of Science Core Collection<br>Current Contents Connect<br>Derwent Innovations Index<br>KCI-Korean Journal Database<br>Medline<br>Russian Science Citation Index<br>SciELO Citation Index	41
TOPIC: ("c1 esterase inhibitor") OR TOPIC: (berinert) OR TOPIC: (cinryze) OR TOPIC: (haegarda) OR TOPIC: (ruconest)<br>Web of Science Core Collection<br>Current Contents Connect<br>Derwent Innovations Index<br>KCI-Korean Journal Database<br>Medline<br>Russian Science Citation Index<br>SciELO Citation Index	1929
**The Cochrane Central Register of Controlled Trials (CENTRAL)**	

MeSH descriptor: [Hemoglobinuria, Paroxysmal] explode all trees or MeSH descriptor: [Atypical Hemolytic Uremic Syndrome] explode all trees or MeSH descriptor: [Myasthenia Gravis] explode all trees or MeSH descriptor: [Graft Rejection] explode all trees or MeSH descriptor: [Delayed Graft Function] explode all trees	2320
("paroxysmal nocturnal hemoglobinuria"):ti,ab,kw OR ("atypical hemolytic uremic syndrome"):ti,ab,kw OR ("myasthenia gravis"):ti,ab,kw OR ("antibody mediated rejection"):ti,ab,kw OR ("ischemia reperfusion injury"):ti,ab,kw OR ("delayed graft function"):ti,ab,kw	2060
(eculizumab):ti,ab,kw OR (soliris):ti,ab,kw	206
(ravulizumab):ti,ab,kw OR (ultomiris):ti,ab,kw OR (alxn1210):ti,ab,kw	27
MeSH descriptor: [Complement C1 Inhibitor Protein] in all MeSH products	73
("c1 esterase inhibitor"):ti,ab,kw OR (berinert):ti,ab,kw OR (cinryze):ti,ab,kw OR (haegarda):ti,ab,kw OR (ruconest):ti,ab,kw	164
**ClinicalTrials.gov, the EU Clinical Trials Register, the United Kingdoms' ISRCTN registry**	
paroxysmal nocturnal hemoglobinuria<br>Also searched for Paroxysmal Hemoglobinuria, PIGA Gene, and Marchiafava Micheli Syndrome in ClinicalTrials.gov	82
atypical hemolytic uremic syndrome	26
myasthenia gravis	97
antibody mediated rejection	59
ischemia reperfusion injury	293
delayed graft function	71
eculizumab<br>Also searched for Soliris in ClinicalTrials.gov	93
ravulizumab<br>Also searched for ALXN 1210 in ClinicalTrials.gov	12
c1 esterase inhibitor<br>Also searched C1 Inhibitor, C1 Inactivator, Complement C1s, Cinryze, Berinert, and SERPING1 in ClinicalTrials.gov	72
**DART Europe E-Theses**	
paroxysmal nocturnal hemoglobinuria	17
atypical hemolytic uremic syndrome	300
myasthenia gravis	240
antibody mediated rejection	138
ischemia reperfusion injury	1340
delayed graft function	119
eculizumab	16
ravulizumab	0
c1 esterase inhibitor	26
berinert	2
ruconest	3
**Open Access Theses and Dissertations (OATD)**	
paroxysmal nocturnal hemoglobinuria	10
atypical hemolytic uremic syndrome	6
myasthenia gravis	107
antibody mediated rejection	37
ischemia reperfusion injury	256
delayed graft function	46
eculizumab	9
ravulizumab	0
c1 esterase inhibitor	27

Berinert	0
ruconest	1
**Oral communications and posters presented in relevant medical society meetings**	
Free manual search<br>American Association of Immunologists Annual Meeting 2006 to 2019<br>European Congress of Immunology 2006, 2009, 2012, 2015, and 2018<br>American Society of Hematology Annual Meeting 2001 to 2019<br>World Congress of the International Society of Hematology 2002, 2005, 2007, 2008, 2010, 2012, 2014, 2016, and 2018<br>American Academy of Neurology Annual Meeting 2002 to 2019<br>World Congress of Neurology 2001, 2005, 2009, 2011, 2013, 2015, 2017, and 2019<br>American Society of Nephrology (ASN) Kidney Week 2003 to 2019<br>International Society of Nephrology (ISN) World Congress of Nephrology 2001, 2003, 2005, 2007, 2009, 2011, 2013, 2015, 2017, and 2019<br>European Renal Association-European Dialysis and Transplant Association (ERA-EDTA) Congress 2003 to 2019<br>American Transplant Congress 2002-2005, 2007-2013, and 2015-2019<br>World Transplant Congress 2006 and 2014	42

References

1. Hillmen, P.; Hall, C.; Marsh, J.C.; Elebute, M.; Bombara, M.P.; Petro, B.E.; Cullen, M.J.; Richards, S.J.; Rollins, S.A.; Mojcik, C.F.; *et al.* Effect of eculizumab on hemolysis and transfusion requirements in patients with paroxysmal nocturnal hemoglobinuria. *N. Engl. J. Med.* **2004**, *350*, 552–559. https://doi.org/10.1056/NEJMoa031688
2. Hill, A.; Hillmen, P.; Richards, S.J.; Elebute, D.; Marsh, J.C.; Chan, J.; Mojcik, C.F.; Rother, R.P. Sustained response and long-term safety of eculizumab in paroxysmal nocturnal hemoglobinuria. *Blood* **2005**, *106*, 2559–2565. https://doi.org/10.1182/blood-2005-02-0564
3. Hillmen, P.; Young, N.S.; Schubert, J.; Brodsky, R.A.; Socié, G.; Muus, P.; Röth, A.; Szer, J.; Elebute, M.O.; Nakamura, R.; *et al.*, The complement inhibitor eculizumab in paroxysmal nocturnal hemoglobinuria. *N. Engl. J. Med.* **2006**, *355*, 1233–1243. https://doi.org/10.1056/NEJMoa061648
4. Brodsky, R.A.; Young, N.S.; Antonioli, E.; Risitano, A.M.; Schrezenmeier, H.; Schubert, J.; Gaya, A; Coyle, L.; de Castro, C.; Fu, C.L.; *et al.* Multicenter phase 3 study of the complement inhibitor eculizumab for the treatment of patients with paroxysmal nocturnal hemoglobinuria. *Blood* **2008**, *111*, 1840–1847. https://doi.org/10.1182/blood-2007-06-094136
5. Hillmen, P.; Muus, P.; Röth, A.; Elebute, M.O.; Risitano, A.M.; Schrezenmeier, H.; Szer, J.; Browne, P.; Maciejewski, J.P.; Schubert, J.; *et al.* Long-term safety and efficacy of sustained eculizumab treatment in patients with paroxysmal nocturnal haemoglobinuria. *Br. J. Haematol.* **2013**, *162*, 62–73. https://doi.org/10.1111/bjh.12347
6. Hillmen, P.; Muus, P.; Dührsen, U.; Risitano, A.M.; Schubert, J.; Luzzatto, L.; Schrezenmeier, H.; Szer, J.; Brodsky, R.A.; Hill, A.; *et al.* Effect of the complement inhibitor eculizumab on thromboembolism in patients with paroxysmal nocturnal hemoglobinuria. *Blood* **2007**, *110*, 4123–4128. https://doi.org/10.1182/blood-2007-06-095646
7. Hillmen, P.; Elebute, M.; Kelly, R.; Urbano-Ispizua, A.; Hill, A.; Rother, R.P.; Khursigara, G.; Fu, C.L.; Omine M.; Browne, P.; *et al.* Long-term effect of the complement inhibitor eculizumab on kidney function in patients with paroxysmal nocturnal hemoglobinuria. *Am. J. Hematol.* **2010**, *85*, 553–559. https://doi.org/10.1002/ajh.21757
8. Kanakura, Y.; Ohyashiki, K.; Shichishima, T.; Okamoto, S.; Ando, K.; Ninomiya, H.; Kawaguchi, T.; Nakao, S.; Nakakuma, H.; Nishimura, J.; *et al.* Safety and efficacy of the terminal complement inhibitor eculizumab in Japanese patients with paroxysmal nocturnal hemoglobinuria: the AEGIS clinical trial. *Int. J. Hematol.* **2011**, *93*, 36–46. https://doi.org/10.1007/s12185-010-0748-9
9. Kanakura, Y.; Ohyashiki, K.; Shichishima, T.; Okamoto, S.; Ando, K.; Ninomiya, H.; Kawaguchi, T.; Nakao, S.; Nakakuma, H.; Nishimura, J.; *et al.* Long-term efficacy and safety of eculizumab in Japanese patients with PNH: AEGIS trial. *Int. J. Hematol.* **2013**, *98*, 406-416. https://doi.org/10.1007/s12185-013-1404-y
10. Lee, J.W.; Sicre de Fontbrune, F.; Wong, Lee Lee L.; Pessoa, V.; Gualandro, S.; Füreder, W.; Ptushkin, V.; Rottinghaus, S.T.; Volles, L.; Shafner, L.; *et al.* Ravulizumab (ALXN1210) vs eculizumab in adult patients with PNH naive to complement inhibitors: the 301 study. *Blood* **2019**, *133*, 530–539. https://doi.org/10.1182/blood-2018-09-876136
11. Kulasekararaj, A.G.; Hill, A.; Rottinghaus, S.T.; Langemeijer, S.; Wells, R.; Gonzalez-Fernandez, F.A.; Gaya, A.; Lee, J.W.; Gutierrez, E.O.; Piatek, C.I.; *et al.* Ravulizumab (ALXN1210) vs eculizumab in C5-inhibitor-experienced adult patients with PNH: the 302 study. *Blood* **2019**, *133*, 540–549. https://doi.org/10.1182/blood-2018-09-876805
12. Schrezenmeier, H.; Muus, P.; Socié, G.; Szer, J.; Urbano-Ispizua, A.; Maciejewski, J.P.; Brodsky, R.A.; Bessler, M.; Kanakura, Y.; Rosse, W.; *et al.* Baseline characteristics and disease burden in patients in the International Paroxysmal Nocturnal Hemoglobinuria Registry. *Haematologica* **2014**, *99*, 922–929. https://doi.org/10.3324/haematol.2013.093161
13. Socié, G.; Schrezenmeier, H.; Muus, P.; Lisukov, I.; Röth, A.; Kulasekararaj, A.; Lee, J.W.; Araten, D.; Hill, A.; Brodsky, R.; *et al.* Changing prognosis in paroxysmal nocturnal haemoglobinuria disease subcategories: an analysis of the International PNH Registry. *Intern. Med. J.* **2016**, *46*, 1044–1053. https://doi.org/10.1111/imj.13160
14. Urbano-Ispizua, A; Muus,P.; Schrezenmeier, H.; Almeida, A.M.; Wilson, A.; Ware, R.E. Different clinical characteristics of paroxysmal nocturnal hemoglobinuria in pediatric and adult patients. *Haematologica* **2017**, *102*, e76–e79. https://doi.org/10.3324/haematol.2016.151852

15. Lee, J.W.; Peffault de Latour, R.; Brodsky, R.A.; Jang, J.H.; Hill, A.; Röth, A.; Schrezenmeier, H.; Wilson, A.; Marantz, J.L.; Maciejewski, J.P. Effectiveness of eculizumab in patients with paroxysmal nocturnal hemoglobinuria (PNH) with or without aplastic anemia in the International PNH Registry. *Am. J. Hematol.* **2019**, *94*, E37–E41. https://doi.org/10.1002/ajh.25334
16. Sakurai, M.; Jang, J.H.; Chou, W.C.; Kim, J.S.; Wilson, A.; Nishimura, J.I.; Chiou, T.J.; Kanakura, Y.; Lee, J.W.; Okamoto, S. Comparative study on baseline clinical characteristics of Asian versus non-Asian patients with paroxysmal nocturnal hemoglobinuria. *Int. J. Hematol.* **2019**, *110*, 411–418. https://doi.org/10.1007/s12185-019-02699-7
17. Ninomiya, H.; Obara, N.; Chiba, S.; Usuki, K.; Nishiwaki, K.; Matsumura, I.; Shichishima, T.; Okamoto, S.; Nishimura, J.-I.; Ohyashiki, K.; *et al.* Interim analysis of post-marketing surveillance of eculizumab for paroxysmal nocturnal hemoglobinuria in Japan. *Int. J. Hematol.* **2016**, *104*, 548–558. https://doi.org/10.1007/s12185-016-2065-4
18. Choi, C.W.; Jang, J.H.; Kim, J.S.; Jo, D.Y.; Lee, J.H.; Kim, S.H.; Kim, Y.K.; Won, J.H.; Chung, J.S.; Kim, H.; *et al.* Efficacy of eculizumab in paroxysmal nocturnal hemoglobinuria patients with or without aplastic anemia: prospective study of a Korean PNH cohort. *Blood Res.* **2017**, *52*, 207–211. https://doi.org/10.5045/br.2017.52.3.207
19. Jang, J.H.; Kim, J.S.; Yoon, S.-S.; Lee, J.-H.; Kim, Y.-K.; Jo, D.-Y.; Chung, J.; Sohn, S.K.; Lee, J.W. Predictive factors of mortality in population of patients with paroxysmal nocturnal hemoglobinuria (PNH): results from a Korean PNH Registry. *J. Korean Med. Sci.* **2016**, *31*, 214–221. https://doi.org/10.3346/jkms.2016.31.2.214
20. Kim, J.S.; Jang, J.H.; Yoon, S.-S.; Lee, J.-H.; Kim, J.-K.; Jo, D.-Y.; Chung, J.S.; Sohn, S.K.; Lee, J.W. Distinct subgroups of paroxysmal nocturnal hemoglobinuria (PNH) with cytopenia: results from South Korean National PNH Registry. *Ann. Hematol.* **2016**, *95*, 125–133. https://doi.org/10.1007/s00277-015-2511-z
21. Kim, J.S.; Cheong, J.W.; Mun, Y.C.; Jang, J.H.; Jo, D.-Y.; Lee, J.W. Clinical implication of renal dysfunction during the clinical course in patients with paroxysmal nocturnal hemoglobinuria: a longitudinal analysis. *Ann. Hematol.* **2019**, *98*, 2273–2281. https://doi.org/10.1007/s00277-019-03735-6
22. Loschi, M.; Porcher, R.; Barraco, F.; Terriou, L.; Mohty, M.; de Guibert, S.; Mahe, B.; Lemal, R.; Dumas, P.-Y.; Etienne, G.; *et al.*, Impact of eculizumab treatment on paroxysmal nocturnal hemoglobinuria: a treatment versus no-treatment study. *Am. J. Hematol.* **2016**, *91*, 366–370. https://doi.org/10.1002/ajh.24278
23. Peffault de Latour, R.; Mary, J.Y.; Salanoubat, C.; Terriou, L.; Etienne, G.; Mohty, M.; Roth, S.; de Guibert, S.; Maury, S.; Cahn, J.Y.; *et al.* Paroxysmal nocturnal hemoglobinuria: natural history of disease subcategories. *Blood* **2008**, *112*, 3099–3106. https://doi.org/10.1182/blood-2008-01-133918
24. Villegas, A.; Núñez, R.; Gaya, A.; Cuevas-Ruiz, M.V.; Bosch, J.M.; Carral, A.; Arrizabalaga, B.; Gómez-Roncero, M.I.; Mora, A.; Bravo, P.; *et al.* Presence of acute and chronic renal failure in patients with paroxysmal nocturnal hemoglobinuria: results of a retrospective analysis from the Spanish PNH Registry. *Ann. Hematol.* **2017**, *96*, 1727–1733. https://doi.org/10.1007/s00277-017-3059-x
25. Muñoz-Linares, C.; Ojeda, E.; Forés, R.; Pastrana, M.; Cabero, M.; Morillo, D.; Bautista, G.; Baños, I.; Monteserín, C.; Bravo, P.; Jaro, E.; *et al.*, Paroxysmal nocturnal hemoglobinuria: a single Spanish center's experience over the last 40 yr. *Eur. J. Haematol.* **2014**, *93*, 309–319. https://doi.org/10.1111/ejh.12346
26. López Rubio, M.; Morado, M.; Gaya, A.; Alonso Rosa, D.; Ojeda, E.; Muñoz, J.A.; Pérez de Mendiguren, B.; Monteagudo, M.D.; Durán, J.M.; Fisac, R.M.; *et al.* [Paroxysmal nocturnal hemoglobinuria therapy with eculizumab: Spanish experience]. *Med. Clin. (Barc.)* **2011**, *137*, 8–13. https://doi.org/10.1016/j.medcli.2010.09.035
27. Wang, H.C.; Kuo, C.Y.; Liu, I.T.; Chen, T.Y.; Chang, Y.H.; Lin, S.J.; Cho, S.F.; Liu, Y.C.; Liu, T.C.; Lin, S.F.; *et al.* Distinct clinical characteristics of paroxysmal nocturnal hemoglobinuria in patients in Southern Taiwan: A multicenter investigation. *Kaohsiung J. Med. Sci.* **2017**, *33*, 405–410. https://doi.org/10.1016/j.kjms.2017.05.011
28. Kelly, R.J.; Hill, A.; Arnold, L.M.; Brooksbank, G.L.; Richards, S.J.; Cullen, M.; Mitchell, L.D.; Cohen, D.R.; Gregory, W.M.; Hillmen, P. Long-term treatment with eculizumab in paroxysmal nocturnal hemoglobinuria: sustained efficacy and improved survival. *Blood* **2011**, *117*, 6786–6792. https://doi.org/10.1182/blood-2011-02-333997
29. Legendre, C.M.; Licht, C.; Muus, P.; Greenbaum, L.A.; Babu, S.; Bedrosian, C.; Bingham, C.; Cohen, D.J.; Delmas, Y.; Douglas, K.; *et al.* Terminal complement inhibitor eculizumab in atypical hemolytic-uremic syndrome. *N. Engl. J. Med.* **2013**, *368*, 2169–2181. https://doi.org/10.1056/NEJMoa1208981

30. Licht, C.; Greenbaum, L.A.; Muus, P.; Babu, S.; Bedrosian, C.L.; Cohen, D.J.; Delmas, Y.; Douglas, K.; Furman, R.R.; Gaber, O.A.; *et al.* Efficacy and safety of eculizumab in atypical hemolytic uremic syndrome from 2-year extensions of phase 2 studies. *Kidney Int.* **2015**, *87*, 1061–1073. https://doi.org/10.1038/ki.2014.423
31. Walle, J.V.; Delmas, Y.; Ardissino, G.; Wang, J.; Kincaid, J. F.; Haller H. Improved renal recovery in patients with atypical hemolytic uremic syndrome following rapid initiation of eculizumab treatment. *J. Nephrol.* **2017**, *30*, 127–134. https://doi.org/10.1007/s40620-016-0288-3
32. Greenbaum, L.A.; Fila, M.; Ardissino, G.; Al-Akash, S.I.; Evans, J.; Henning, P.; Lieberman, K.V.; Maringhini, S.; Pape, L.; Rees, L.; *et al.* Eculizumab is a safe and effective treatment in pediatric patients with atypical hemolytic uremic syndrome. *Kidney Int.* **2016**, *89*, 701–711. https://doi.org/10.1016/j.kint.2015.11.026
33. Fakhouri, F.; Hourmant, M.; Campistol, J.M.; Cataland, S.R.; Espinosa, M.; Gaber, A.O.; Menne, J.; Minetti, E.E.; Provôt, F.; Rondeau, E.; *et al.* Terminal complement inhibitor eculizumab in adult patients with atypical hemolytic uremic syndrome: a single-arm, open-label trial. *Am. J. Kidney Dis.* **2016**, *68*, 84–93. https://doi.org/10.1053/j.ajkd.2015.12.034
34. Menne, J.; Delmas, Y.; Fakhouri, F.; Licht, C.; Lommelé, Å.; Minetti, E.E.; Provôt, F.; Rondeau, E.; Sheerin, N.S.; Wang, J.; *et al.* Outcomes in patients with atypical hemolytic uremic syndrome treated with eculizumab in a long-term observational study. *BMC Nephrol.* **2019**, *20*, 125. https://doi.org/10.1186/s12882-019-1314-1
35. Menne, J.; Delmas, Y.; Fakhouri, F.; Kincaid, J.F.; Licht, C.; Minetti, E.E.; Mix, C.; Provôt, F.; Rondeau, E.; Sheerin, N.S.; *et al.* Eculizumab prevents thrombotic microangiopathy in patients with atypical haemolytic uraemic syndrome in a long-term observational study. *Clin. Kidney J.* **2018**, *12*, 196–205. https://doi.org/10.1093/ckj/sfy035
36. Rondeau, E.; Scully, M.; Ariceta, G.; Barbour, T.D.; Cataland, S.R.; Heyne, N.; Miyakawa, Y.; Ortiz, D.; Swenson, E.D.; Vallee, M.; *et al.* TH-PO800: Efficacy and safety of the Long-acting C5-inhibitor ravulizumab in adult patients with atypical hemolytic uremic syndrome (aHUS). American Society of Nephrology (ASN) Kidney Week 2019, Washington, November 5–10, 2019. https://www.asn-online.org/education/kidneyweek/2019/program-abstract.aspx?controlId=3230505
37. Licht, C.; Ardissino, G.; Ariceta, G.; Cohen, D.; Cole, J.A.; Gasteyger, C.; Greenbaum, L.A.; Johnson, S.; Ogawa, M.; Schaefer, F.; *et al.* The Global aHUS Registry: methodology and initial patient characteristics. *BMC Nephrol.* **2015**, *16*, 207. https://doi.org/10.1186/s12882-015-0195-1
38. Rondeau, E.; Cataland, S.R.; Al-Dakkak, I.; Miller, B.; Webb, N.J.A.; Landau, D. Eculizumab safety: five-year experience from the Global Atypical Hemolytic Uremic Syndrome Registry. *Kidney Int. Rep.* **2019**, *4*, 1568–1576. https://doi.org/10.1016/j.ekir.2019.07.016
39. Schaefer, F.; Ardissino, G.; Ariceta, G.; Fakhouri, F.; Scully, M.; Isbel, N.; Lommelé, Å.; Kupelian, V.; Gasteyger, C.; Greenbaum, L.A.; *et al.*, Clinical and genetic predictors of atypical hemolytic uremic syndrome phenotype and outcome. *Kidney Int.* **2018**, *94*, 408–418. https://doi.org/10.1016/j.kint.2018.02.029
40. Leanne, C.; Rondeau, E.; Ardissino, G.; Caby-Tosi, M.-P.; Al-Dakkak, I.; Fakhouri, F.; Miller, B.; Scully, M. SP075: Pregnancy outcomes in patients enrolled in the Global aHUS Registry. 56th European Renal Association – European Dialysis and Transplant Association (ERA-EDTA) Congress, Budapest, June 13–16, 2019. http://mediatheque.cyim.com/mediatheque/media.aspx?mediaId=65067&channel=60398
41. Ito, S.; Hidaka, Y.; Inoue, N.; Kaname, S.; Kato, H.; Matsumoto, M.; Miyakawa, Y.; Mizuno, M.; Okada, H.; Shimono, A.; *et al.* Safety and effectiveness of eculizumab for pediatric patients with atypical hemolytic-uremic syndrome in Japan: interim analysis of post-marketing surveillance. *Clin. Exp. Nephrol.* **2019**, *23*, 112–121. https://doi.org/10.1007/s10157-018-1610-2
42. Kato, H.; Miyakawa, Y.; Hidaka, Y.; Inoue, N.; Ito, S.; Kagami, S.; Kaname, S.; Matsumoto, M.; Mizuno, M.; Matsuda, T.; *et al.* Safety and effectiveness of eculizumab for adult patients with atypical hemolytic-uremic syndrome in Japan: interim analysis of post-marketing surveillance. *Clin. Exp. Nephrol.* **2019**, *23*, 65–75. https://doi.org/10.1007/s10157-018-1609-8
43. Ito, N.; Hataya, H; Saida, K.; Amano, Y.; Hidaka, Y.; Motoyoshi, Y.; Ohta, T.; Yoshida, Y.; Terano, C.; Iwasa, T.; *et al.* Efficacy and safety of eculizumab in childhood atypical hemolytic uremic syndrome in Japan. *Clin. Exp. Nephrol.* **2016**, *20*, 265–272. https://doi.org/10.1007/s10157-015-1142-y
44. Mallett, A.; Hughes, P.; Szer, J.; Tuckfield, A.; Van Eps, C.; Cambell, S.B.; Hawley, C.; Burke, J.; Kausman, J.; Hewitt, I.; *et al.* Atypical haemolytic uraemic syndrome treated with the complement inhibitor eculizumab: the experience of the Australian compassionate access cohort. *Intern. Med J.* **2015**, *45*, 1054–1065. https://doi.org/10.1111/imj.12864

45. de Andrade, L.G.M.; Contti, M.M.; Nga, H.S.; Bravin, A.M.; Takase, H.M.; Viero, R.M.; da Silva, T.N.; Chagas, K.N.; Palma, L.M.P. Long-term outcomes of the atypical hemolytic uremic syndrome after kidney transplantation treated with eculizumab as first choice. *PLoS One* **2017**, *12*, e0188155. https://doi.org/10.1371/journal.pone.0188155
46. Besbas, N.; Gulhan, B.; Soylemezoglu, O.; Ozcakar, Z.B.; Korkmaz, E.; Hayran, M.; Ozaltin, F. Turkish pediatric atypical hemolytic uremic syndrome registry: initial analysis of 146 patients. *BMC Nephrol.* **2017**, *18*, 6. https://doi.org/10.1186/s12882-016-0420-6
47. Gediz, F.; Payzin, B.K.; Ecemis, S.; Güler, N.; Yilmaz, A.F.; Topcugil, F.; Berdeli, A. Efficacy and safety of eculizumab in adult patients with atypical hemolytic uremic syndrome: A single center experience from Turkey. *Transfus. Apher. Sci.* **2016**, *55*, 357–362. https://doi.org/10.1016/j.transci.2016.09.017
48. Yenerel, M.N.; Aktan, M.; Yildiz, A.; Caliskan, Y.; Nalcaci, M. 311-II-4: The importance of Eculizumab in patients with atypical Hemolytic Uremic Syndrome: single center experience. 56th American Society of Hematology (ASH) annual meeting, San Francisco, December 6–9, 2014. https://ashpublications.org/blood/article/124/21/2777/95757/The-Importance-of-Eculizumab-in-Patients-with
49. Le Clech, A.; Simon-Tillaux, N.; Provôt, F.; Delmas; Y.; Vieira-Martins, P.; Limou, S.; Halimi, J.M.; Le Quintrec, M.; Lebourg, L.; Grangé, S.; *et al.* Atypical and secondary hemolytic uremic syndromes have a distinct presentation and no common genetic risk factors. *Kidney Int.* **2019**, *95*, 1443–1452. https://doi.org/10.1016/j.kint.2019.01.023
50. Fakhouri, F.; Delmas, Y.; Provot, F.; Barbet, C.; Karras, A.; Makdassi, R.; Courivaud, C.; Rifard, K.; Servais, A.; Allard, C.; *et al.* Insights from the use in clinical practice of eculizumab in adult patients with atypical hemolytic uremic syndrome affecting the native kidneys: an analysis of 19 cases. *Am. J. Kidney Dis.* **2014**, *63*, 40–48 (2014). https://doi.org/10.1053/j.ajkd.2013.07.011
51. Fremeaux-Bacchi, V.; Fakhouri, F.; Garnier, A.; Bienaimé, F.; Dragon-Durey, M.A.; Ngo, S.; Moulin, B.; Servais, A.; Provot, F.; Rostaing, L.; *et al.* Genetics and outcome of atypical hemolytic uremic syndrome: a nationwide French series comparing children and adults. *Clin. J. Am. Soc. Nephrol.* **2013**, *8*, 554–562. https://doi.org/10.2215/CJN.04760512
52. de Fontbrune, F.S.; Galambrun, C.; Sirvent, A.; Huynh, A.; Faguer, S.; Nguyen, S.; Bay, J.O.; Neven, B.; Moussi, J.; Simon, L.; *et al.* Use of eculizumab in patients with allogeneic stem cell transplant-associated thrombotic microangiopathy: a study from the SFGM-TC. *Transplantation* **2015**, *99*, 1953–1959. https://doi.org/10.1097/TP.0000000000000601
53. Le Quintrec, M.; Lionet, A.; Kamar, N.; Karras, A.; Barbier, S.; Buchler, M.; Fakhouri, F.; Provost, F.; Fridman, W.H.; Thervet, E.; *et al.* Complement mutation-associated de novo thrombotic microangiopathy following kidney transplantation. *Am. J. Transplant.* **2008**, *8*, 1694–1701. https://doi.org/10.1111/j.1600-6143.2008.02297.x
54. Zuber, J.; Le Quintrec, M.; Krid, S.; Bertoye, C.; Gueutin, V.; Lahoche, A.; Heyne, N.; Ardissino, G.; Chatelet, V.; Noël, L.-H.; *et al.* Eculizumab for atypical hemolytic uremic syndrome recurrence in renal transplantation. *Am. J. Transplant.* **2012**, *12*, 3337–3354. https://doi.org/10.1111/j.1600-6143.2012.04252.x
55. Servais, A.; Noël, L.H.; Roumenina, L.T.; Le Quintrec, M.; Ngo, S.; Dragon-Durey, M.A.; Macher, M.A.; Zuber, J.; Karras, A.; Provot, F.; *et al.* Acquired and genetic complement abnormalities play a critical role in dense deposit disease and other C3 glomerulopathies. *Kidney Int.* **2012**, *82*, 454–464. https://doi.org/10.1038/ki.2012.63
56. Cavero, T.; Rabasco, C.; López, A.; Román, E.; Ávila, A.; Sevillano, Á.; Huerta, A.; Rojas-Rivera, J.; Fuentes, C.; Blasco, M.; *et al.* Eculizumab in secondary atypical haemolytic uraemic syndrome. *Nephrol. Dial. Transplant.* **2017**, *32*, 466–474. https://doi.org/10.1093/ndt/gfw453
57. Huerta, A.; Arjona, E.; Portoles, J.; Lopez-Sanchez, P.; Rabasco, C.; Espinosa, M.; Cavero, T.; Blasco, M.; Cao, M.; Manrique, J.; *et al.* A retrospective study of pregnancy-associated atypical hemolytic uremic syndrome. *Kidney Int.* **2018**, *93*, 450–459. https://doi.org/10.1016/j.kint.2017.06.022
58. Cavero, T.; Rodríguez de Córdoba, S.; Praga, M. 121: ¿Microangiopatía trombótica asociada a hipertensión o a hiperactividad del complemento? Implicaciones diagnósticas y terapéuticas. XLVIII Congreso de la Sociedad Española de Nefrología y IX Congreso Iberoamericano de Nefrología, Madrid, November 16–19, 2018. https://www.senefro.org/modules.php?name=noticias&d_op=view&idnew=1124
59. Sheerin, N.S.; Kavanagh, D.; Goodship, T.H.J.; Johnson, S. A national specialized service in England for atypical haemolytic uraemic syndrome-the first year's experience. *QJM* **2016**, *109*, 27–33. https://doi.org/10.1093/qjmed/hcv082

60. Brocklebank, V.; Johnson, S.; Sheerin, T.P.; Marks, S.D.; Gilbert, R.D.; Tyerman, K.; Kinoshita, M.; Awan, A.; Kaur, A.; Webb, N.; *et al.*, Factor H autoantibody is associated with atypical hemolytic uremic syndrome in children in the United Kingdom and Ireland. *Kidney Int.* **2017**, *92*, 1261–1271. https://doi.org/10.1016/j.kint.2017.04.028
61. Howard, J.F.Jr; Barohn, R.J.; Cutter, G.R.; Freimer, M.; Juel, V.C.; Mozaffar, T.; Mellion, M.L.; Benatar, M.G.; Farrugia, M.E.; Wang, J.J.; *et al.* A randomized, double-blind, placebo-controlled phase II study of eculizumab in patients with refractory generalized myasthenia gravis. *Muscle Nerve* **2013**, *48*, 76–84. https://doi.org/10.1002/mus.23839
62. Howard, J.F.Jr.; Utsugisawa, K.; Benatar, M.; Murai, H.; Barohn, R.J.; Illa, I.; Jacob, S.; Vissing, J.; Burns, T.M.; Kissel, J.T.; *et al.* Safety and efficacy of eculizumab in anti-acetylcholine receptor antibody-positive refractory generalised myasthenia gravis (REGAIN): a phase 3, randomised, double-blind, placebo-controlled, multicentre study. *Lancet Neurol.* **2017**, *16*, 976–986. https://doi.org/10.1016/S1474-4422(17)30369-1
63. Muppidi, S.; Utsugisawa, K.; Benatar, M.; Murai, H.; Barohn, R.J.; Illa, I.; Jacob, S.; Vissing, J.; Burns, T.M.; Kissel, J.T.; *et al.* Long-term safety and efficacy of eculizumab in generalized myasthenia gravis. *Muscle Nerve* **2019**, *60*, 14–24. https://doi.org/10.1002/mus.26447
64. Viglietti, D.; Gosset, C.; Loupy, A.; Deville, L.; Verine, J.; Zeevi, A.; Glotz, D.; Lefaucheur, C. C1 Inhibitor in acute antibody-mediated rejection nonresponsive to conventional therapy in kidney transplant recipients: a pilot study. *Am. J. Transplant.* **2016**, *16*, 1596–1603. https://doi.org/10.1111/ajt.13663
65. Montgomery, R.A.; Orandi, B.J.; Racusen, L.; Jackson, A.M.; Garonzik-Wang, J.M.; Shah, T.; Woodle, E.S.; Sommerer, C.; Fitts, D.; Rockich, K.; *et al.* Plasma-derived C1 esterase inhibitor for acute antibody-mediated rejection following kidney transplantation: results of a randomized double-blind placebo-controlled pilot study. *Am. J. Transplant.* **2016**, *16*, 3468–3478. https://doi.org/10.1111/ajt.13871
66. Stegall, M.D.; Diwan, T.; Raghavaiah, S.; Cornell, L.D.; Burns, J.; Dean, P.G.; Cosio, F.G.; Gandhi, M.J.; Kremers, W.; Gloor, J.M. Terminal complement inhibition decreases antibody-mediated rejection in sensitized renal transplant recipients. *Am. J. Transplant.* **2011**, *11*, 2405–2413. https://doi.org/10.1111/j.1600-6143.2011.03757.x
67. Vo, A.A.; Zeevi, A.; Choi, J.; Cisneros, K.; Toyoda, M.; Kahwaji, J.; Peng, A.; Villicana, R.; Puliyanda, D.; Reinsmoen, N.; *et al.* A phase I/II placebo-controlled trial of C1-inhibitor for prevention of antibody-mediated rejection in HLA sensitized patients. *Transplantation* **2015**, *99*, 299–308. https://doi.org/10.1097/TP.0000000000000592
68. Vo, A.A.; Choi, J.; Kahwaji, J.; Puliyanda, D.; Peng, A.; Villicana, R.; Jordan, S. A194: Long-term analysis of a placebo-controlled trial of C1-INH for prevention of antibody-mediated rejection. 2015 American Transplant Congress, Philadelphia, May 3–6, 2015. https://atcmeetingabstracts.com/abstract/long-term-analysis-of-a-placebo-controlled-trial-of-c1-inh-for-prevention-of-antibody-mediated-rejection/
69. Vo, A.A.; Choi, J.; Peng, A.; Lim, K.; Varanasi, L.; Najjar, R.; Huang, E.; Puliyanda, D.; Jordan, S. A50: Update of a placebo-controlled trial of C1 esterase inhibitor for prevention of antibody mediated rejection (ABMR) in highly-HLA sensitized patients. 2017 American Transplant Congress, Chicago, April 29–May 3, 2015. https://atcmeetingabstracts.com/abstract/update-of-a-placebo-controlled-trial-of-c1-esterase-inhibitor-for-prevention-of-antibody-mediated-rejection-abmr-in-highly-hla-sensitized-patients/
70. Marks, W.H.; Mamode, N.; Montgomery, R.A.; Stegall, M.D.; Ratner, L.E.; Cornell, L.D.; Rowshani, A.T.; Colvin, R.B.; Dain, B.; Boice, J.A.; *et al.* Safety and efficacy of eculizumab in the prevention of antibody-mediated rejection in living-donor kidney transplant recipients requiring desensitization therapy: A randomized trial. *Am. J. Transplant.* **2019**, *19*, 2876–2888. https://doi.org/10.1111/ajt.15364
71. Glotz, D.; Russ, G.; Rostaing, L.; Legendre, C.; Tufveson, G.; Chadban, S.; Grinyó, J.; Mamode, N.; Rigotti, P.; Couzi, L.; *et al.* Safety and efficacy of eculizumab for the prevention of antibody-mediated rejection after deceased-donor kidney transplantation in patients with preformed donor-specific antibodies. *Am. J. Transplant.* **2019**, *19*, 2865–2875. https://doi.org/10.1111/ajt.15397
72. Kaabak, M.; Babenko, N.; Shapiro, R.; Zokoyev, A.; Dymova, O.; Kim, E. A prospective randomized, controlled trial of eculizumab to prevent ischemia-reperfusion injury in pediatric kidney transplantation. *Pediatr. Transplant.* **2018**, *22*, e13129. https://doi.org/10.1111/petr.13129
73. Jordan, S.C.; Choi, J.; Aubert, O.; Haas, M.; Loupy, A.; Huang, E.; Peng, A.; Kim, I.; Louie, S.; Ammerman, N.; *et al.*, A phase I/II, double-blind, placebo-controlled study assessing safety and efficacy of C1 esterase inhibitor for prevention of delayed graft function in deceased donor kidney transplant recipients. *Am. J. Transplant.* **2018**, *18*, 2955–2964. https://doi.org/10.1111/ajt.14767

74. Schröppel, B.; Akalin, E.; Baweja, M.; Bloom, R.D.; Florman, S.; Goldstein, M.; Haydel, B.; Hricik, D.E.; Kulkarni, S.; Levine, M.; *et al.*, Peritransplant eculizumab does not prevent delayed graft function in deceased donor kidney transplant recipients: Results of two randomized controlled pilot trials. *Am. J. Transplant.* **2020**, *20*, 1–9. https://doi.org/10.1111/ajt.15580
75. Prevention of Delayed Graft Function Using Eculizumab Therapy (PROTECT Study). ClinicalTrials.gov. https://clinicaltrials.gov/ct2/show/NCT02145182
